# S-Propargyl-Cysteine, a Novel Hydrogen Sulfide Donor, Inhibits Inflammatory Hepcidin and Relieves Anemia of Inflammation by Inhibiting IL-6/STAT3 Pathway

**DOI:** 10.1371/journal.pone.0163289

**Published:** 2016-09-20

**Authors:** Minjun Wang, Wenbo Tang, Hong Xin, Yi Zhun Zhu

**Affiliations:** 1 Shanghai Key Laboratory of Bioactive Small Molecules, Department of Pharmacology, School of Pharmacy, Fudan University, Shanghai, 201203, China; 2 Department of Medical Oncology, Fudan University Shanghai Cancer Center, Shanghai, 200032, China; 3 Department of Pharmacology, School of Pharmacy, Macau University of Science & Technology, Macau, China; Medizinische Fakultat der RWTH Aachen, GERMANY

## Abstract

Anemia of inflammation (AI) is clinically prevalent and greatly threatens public health. Traditional remedies have raised controversy during clinical practice, calling for alternative therapies. We have recently found that hydrogen sulfide (H_2_S) inhibits inflammatory hepcidin, the critical mediator of AI. However, due to the chemical property of H_2_S, there remains an urgent need for a stable H_2_S donor in AI treatment. Here we reported that S-propargyl-cysteine (SPRC), a novel water-soluble H_2_S donor, suppressed hepatic hepcidin and corrected hypoferremia induced by lipopolysaccharide. The effects of SPRC were reversed by inhibition of cystathionine γ-lyase, one of the major endogenous H_2_S synthases. Moreover, SPRC reduced serum hepcidin, improved transferrin saturation, and maintained erythrocyte membrane integrity in a chronic mouse AI model. Consistently, splenomegaly was ameliorated and splenic iron accumulation relieved. Mechanism study indicated that serum IL-6 content and hepatic *Il-6* mRNA were decreased by SPRC, in parallel with reduced hepatic JAK2/STAT3 activation. On the whole, our data reveal the inhibition of inflammatory hepcidin by SPRC, and suggest SPRC as a potential remedy against AI.

## Introduction

Anemia of inflammation (AI) is the second most prevalent anemia after anemia of iron deficiency [1]. Since AI is often accompanied by chronic diseases, such as cancer, chronic infections, and auto-immune syndrome, it is also named as anemia of chronic diseases. Despite the fact that AI is relatively mild (hemoglobin levels between 90–120 g/L), mounting evidences have revealed its relation to poor prognosis and increased mortality [2, 3]. In addition, concerns have arisen with respect to the effectiveness and safety of conventional therapies [4, 5], spurring the search for alternative remedies for AI.

It is currently well established that AI is a chronic inflammatory disease in nature. Hepcidin, a hepatic iron-regulatory hormone, acts as the prominent modulator of AI [6]. Incremental hepcidin production is observed in AI patients [7], while inhibition of hepcidin protects mice from AI [8]. Previous studies have demonstrated that inflammatory hepcidin is induced by IL-6-mediated hepatic JAK2/STAT3 activation [9]. Phosphorylated STAT3 dimer directly binds to hepcidin promoter and activates its transcription [10]. Increased hepcidin levels suppress dietary iron absorption and promote iron retention in spleen and liver, thus lower circulating iron content and inhibit erythropoiesis. Therefore, inhibiting inflammatory hepcidin by blocking IL-6/JAK2/STAT3 pathway is believed as a novel approach to treating AI.

Hydrogen sulfide (H_2_S) is the third gasotransmitter and tightly involved in various pathophysiological conditions [11, 12]. H_2_S is endogenously produced by at least 3 enzymes, including cystathionine γ-lyase (CSE), and exerts regulatory effects in inflammation [13]. In our recent work, we reported for the first time that sodium hydrosulfide (NaHS), an exogenous H_2_S donor, inhibited inflammatory hepcidin and relieved hypoferremia induced by acute inflammation [14]. However, NaHS releases H_2_S in a robust and transient manner [15], thus is more a pharmacological reagent than a potential remedy. This prompted us to investigate other sustained-releasing H_2_S donors.

S-propargyl-cysteine (SPRC) is an analog of S-allyl-cysteine (SAC), an H_2_S donor originally derived from garlic extract [16]. Similar to SAC, SPRC serves as a substrate of CSE and increases endogenous H_2_S production. Compared with NaHS, SPRC is more chemically stable and releases H_2_S in a slower and more sustained manner, with a half-life of about 3 h in rats [17], and about 16 h in Beagle dogs [18]. As an H_2_S donor, SPRC elicits extensive regulation on pathological conditions. Many studies have been published reporting the protective effects of SPRC in ischemia/hypoxia injury [19], angiogenesis [20], cognitive impairment [21], and acute pancreatitis [22]. But so far no research has been done regarding its effects on inflammatory hepcidin and iron balance. Moreover, there remains a gap of knowledge concerning the effects of H_2_S donors on chronic AI.

In the present study, we aimed to evaluate the effects of SPRC on inflammatory hepcidin, and assessed its therapeutic potential against AI. We discovered that SPRC, by inhibiting IL-6/JAK2/STAT3 pathway, could reduce inflammatory hepcidin activation *in vivo* both in acute and chronic AI models. Our results indicate SPRC as a potential drug, and throw light on novel treatment strategy for AI.

## Materials and Methods

### Compounds and reagents

**Fig 1:**
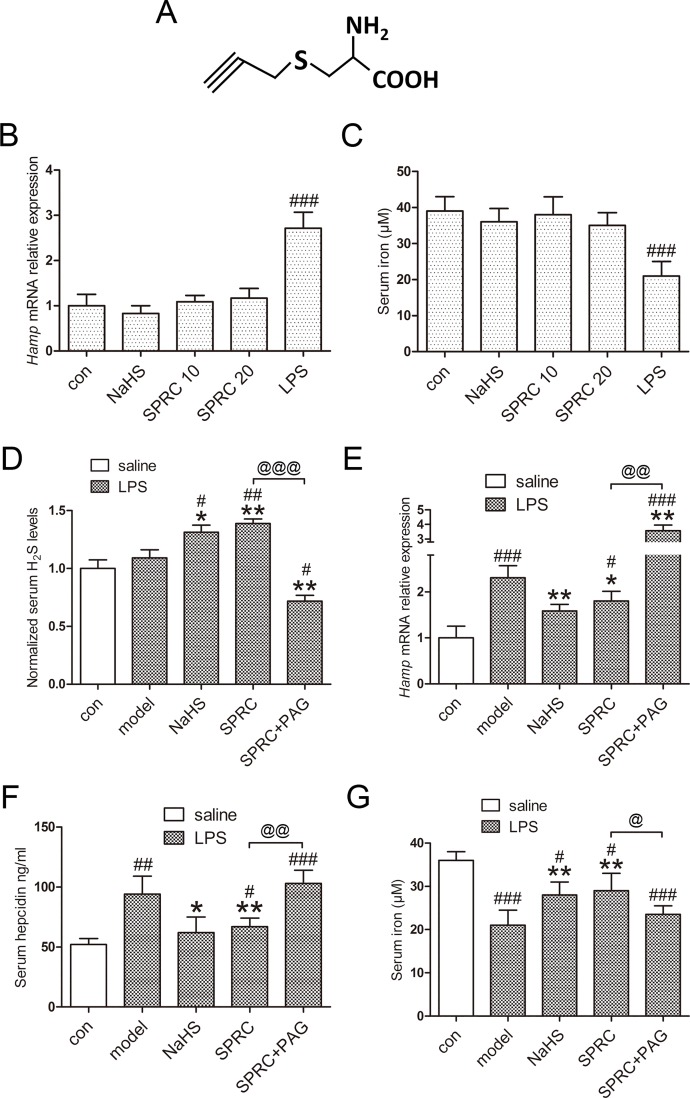
SPRC inhibits inflammatory hepcidin and hypoferremia induced by LPS. (A) Chemical structure of SPRC. (B-C) SPRC and NaHS showed no effects on hepatic and serum hepcidin in the absence of LPS (n = 5). (D) SPRC and NaHS increased serum H_2_S content, as opposed to PAG (n = 5). (E-F) Hepatic hepcidin mRNA and serum hepcidin levels were suppressed by SPRC and NaHS, while PAG abolished the effects of SPRC (n = 5). (G) Consistent results were obtained with serum iron levels (n = 5). Data are presented as the mean ± SEM. ^###^ *p* < 0.001 compared with the control group; * *p* < 0.01, ** *p* < 0.01 compared with the LPS group; ^@^ *p* < 0.05, ^@@^ *p* < 0.01, ^@@@^ *p* < 0.001.

SPRC (Fig 1A) was synthesized by the reaction of L-cysteine and propargyl bromide, followed by recrystallization to reach 99% purity as described previously [19]. NaHS, lipopolysaccharide (LPS), propargylglycine (PAG), turpentine, and 1, 10-phenanthroline monohydrate were all purchased from Sigma-Aldrich, St. Louis, MO, USA.

### Animals

All animal experimental protocols complied with the Animal Management Rules of the Ministry of Health of the People’s Republic of China, and were approved by the Animal Care Committee of Fudan University (No. 13111243). Mice were sacrificed after anesthesia with pentobarbital sodium. Every effort was made to ameliorate animal sufferings during the experiments.

Eight-week-old male C57BL/6 mice (about 20 g/each) were purchased from Sippr-bk Experimental Animal Center, Shanghai, China. Mice were housed under specific pathogen-free (SPF) rooms at 25°C and maintained under a 12-h/12-h light/dark cycle with *ad libitum* access to food and water.

### Acute LPS model and chronic turpentine model

To evaluate the effects of SPRC on hepatic hepcidin and hypoferremia during acute inflammation, C57BL/6 mice were *i*.*p*. injected with 10 mg/kg SPRC daily for 1 week. Meanwhile, PAG (15 mg/kg, *i*.*p*.) was applied with SPRC to inhibit CSE-mediated H_2_S production, while NaHS (6 mg/kg, *i*.*p*.) served as a positive control for 3 days. The doses for PAG and SPRC were chosen by reference to previous reports [22, 23]. One hour after the last injection, mice were challenged with 0.5 mg/kg LPS (*i*.*p*.) and sacrificed 6 h later after anesthesia with pentobarbital sodium. Normal saline was used as vehicle control.

For turpentine model, C57BL/6 mice were subcutaneously injected with turpentine (100 μl/20 g weight) once a week for 4 weeks to induce chronic AI. SPRC (*i*.*p*., 10/20 mg/kg) was administrated twice a week, starting from the second injection of turpentine. Mice were sacrificed on the fifth week after anesthesia with pentobarbital sodium. PAG significantly increased mortality in this model, thus was not applied here.

### RNA isolation and real-time qRT-PCR

Total RNA from mouse livers was extracted using RNAiso Plus (TAKARA Bio, China) according to the manufacturer’s instructions. Reverse transcription of total RNA was carried out with PrimeScript™ RT Master Mix (Perfect Real Time, TAKARA Bio, China). Real-time qPCR was performed with Bio-Rad CFX ConnectTM Real-Time PCR System and primers as follows: murine *β-actin* forward 5’-TGTTACCAACTGGGACGACA-3’, reverse 5’-GGTGTTGAAGGTCTCAAA-3’; murine *Hamp* forward 5’-AGAGCTGCAGCCTTTGCAC-3’, reverse 5’-GAAGATGCAGATGGGGAAGT-3’; murine *Il-6* forward 5’-TGTGCAATGGCAATTCTGAT-3’, reverse 5’-CCAGAGGAAATTTTCAATAGGC-3’; murine suppressor of cytokine signaling 3 *(Socs3)* forward 5’-TGCGCCTCAAGACCTTCAG-3’, reverse 5’-GCTCCAGTAGAATCCGCTCTC-3’; murine serum amyloid A 2 *(Saa2)* forward 5’-TGGCTGGAAAGATGGAGACAA-3’, reverse 5’-AAAGCTCTCTCTTGCATCACTG-3’; Specificity of all PCR products was confirmed by melting curve analysis.

### Immunoblot analysis

For SDS-PAGE, mouse liver tissues were homogenized in RIPA lysis buffer (50 mM Tris-HCl, 150 mM NaCl, 5 mM EDTA, 1% Triton X-100, 1% sodium deoxycholate and 0.1% SDS, pH 7.4) containing protease and phosphatase inhibitor cocktails (Sigma-Aldrich). Fifty micrograms of protein were subjected to SDS-PAGE gels for each sample, and transferred to PVDF membranes (Millipore, Bedford, MA, USA) followed by blocking with 5% skim milk and incubation with primary antibodies overnight at 4°C. Antibodies to total JAK2, total and phospho-STAT3 (Tyr705) were obtained from Cell Signaling Technology Beverly, MA USA. Antibody to phospho-JAK2 (Tyr221) was acquired from Bioworld Technology, Louis Park, MN, USA. Antibody to GAPDH was obtained from Proteintech Group, Chicago, IL, USA. Immunoreactive proteins were visualized and quantified by densitometry using a Bio-Rad Image Lab system. GAPDH served as the loading control.

### Serum iron, hepcidin and IL-6 analysis

Mouse blood samples were collected in non-heparinized tubes, allowed to stand for 2 h at room temperature, and then centrifuged at 3000 rpm for 10 min to separate serum. Serum iron content and total iron-binding capacity (TIBC) were determined by commercial kits according to protocols described by Jiancheng Bioengineering Institute, China. Transferrin saturation was calculated as serum iron/TIBC × 100%.

Murine serum hepcidin and IL-6 were quantified using ELISA kits from USCN (China) and Boatman (China), respectively.

### Quantification of H_2_S concentration

H_2_S determination was conducted by the methylene blue method as described previously [23].

### Tissue non-heme iron analysis

Non-heme iron of mouse spleen was determined using 1, 10-Phenanthroline monohydrate as described previously [14]. In brief, dried spleen tissues were digested in acid solutions (3M hydrochloric acid and 10% trichloroacetic acid). After centrifuge, the supernatant was mixed with 1, 10-Phenanthroline, and the absorbance at 510 nm was measured using a spectrophotometer.

### Wright-Giemsa staining and Perl’s Prussian blue staining

Peripheral blood smears were performed with 3 μl fresh EDTA-treated whole blood, and stained with Wright-Giemsa solution (Yeasen Biotech, China). The slides were then visualized with Zeiss Axio Scope A1 system.

Mouse spleen tissues were fixed in 4% formalin PBS solution, embedded in paraffin wax, sectioned, and stained with Perl’s Prussian blue solution for 30 min at room temperature. A neutral red counterstain was then applied to provide a contrasting background. Images were captured using Zeiss Axio Scope A1 system.

### Statistical analysis

Data are expressed as the mean ± SEM. Statistical analysis was performed with one-way ANOVA followed by Turkey’s test. A two-tailed *p* < 0.05 was considered statistically significant.

## Results

### SPRC reduces inflammatory hepcidin expression by suppressing IL-6/JAK2/STAT3 pathway in vivo

In the absence of LPS challenge, treatment of SPRC and NaHS alone elicited no significant effects on hepatic hepcidin and serum iron (Fig 1B and 1C). As demonstrated in Fig 1D, pretreatment of SPRC and NaHS significantly increased serum H_2_S levels, as opposed to SPRC + PAG. By performing qRT-PCR and ELISA, we analyzed hepatic hepcidin mRNA expression and serum hepcidin levels, and found that both SPRC and NaHS inhibited hepcidin activation, which was exacerbated by PAG (Fig 1E and 1F). Consistent results were observed with serum iron concentration (Fig 1G).

**Fig 2:**
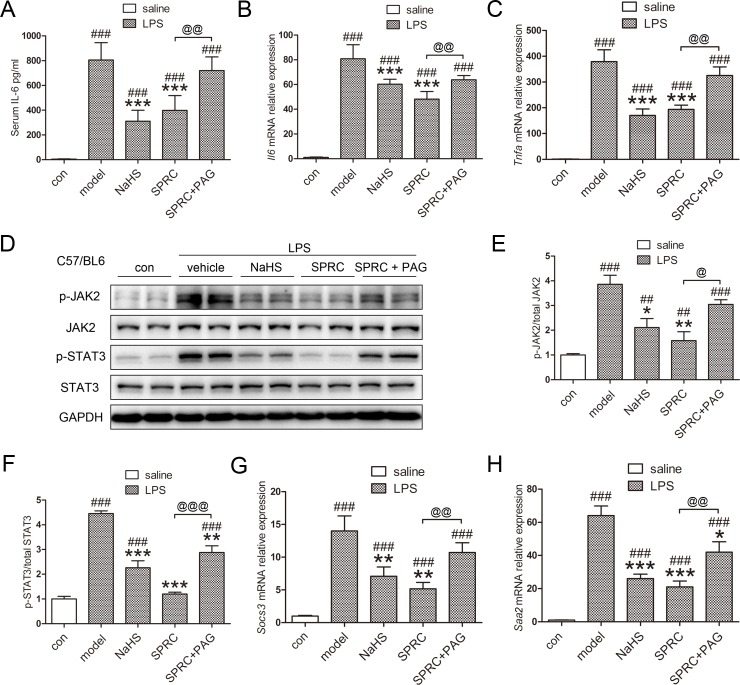
SPRC reduces inflammatory hepcidin activation by inhibiting IL-6/JAK2/STAT3 pathway. (A) SPRC and NaHS markedly decreased serum IL-6 levels induced by LPS (n = 5). (B-D) Hepatic *Il-6*, *Tnfa* mRNA levels and JAK2/STAT3 phosphorylation were induced by LPS, and suppressed by SPRC and NaHS. Moreover, PAG diminished the effects of SPRC (n = 5). (E-F) Densitometry analysis of Fig 2D. (G-H) Hepatic mRNA levels of *Socs3* and *Saa2*, two target genes of STAT3 (n = 5). Representative immunoblots are presented. Whole uncropped images of Fig 2D are shown in S1 Fig. Data are presented as the mean ± SEM. ^###^ *p* < 0.001 compared with the control group; * *p* < 0.05, ** *p* < 0.01, *** *p* < 0.001, compared with the LPS group; ^@^ *p* < 0.05, ^@@^ *p* < 0.01, ^@@@^ *p* < 0.001.

Considering the dominant role IL-6/STAT3 plays in inflammatory hepcidin induction, we then assessed whether SPRC modulated IL-6 production and JAK2/STAT3 pathway. As expected, SPRC and NaHS markedly reduced serum IL-6 levels by more than 50%, which was abrogated by PAG (Fig 2A). To better evaluate the regulation of local inflammation in the liver, we examined hepatic *Il-6* and *Tnfa* mRNA expression and got similar results (Fig 2B and 2C). The minor differences between serum IL-6 content and hepatic *Il-6* levels could be attributed to different systemic and local inflammatory status. Moreover, SPRC successfully suppressed hepatic JAK2/STAT3 phosphorylation (Fig 2D–2F). Consistent data were obtained with the expression of suppressor of cytokine signaling 3 (*Socs3*) and serum amyloid A 2 (*Saa2*), two target genes of STAT3 (Fig 2G and 2H). These data indicate that SPRC, as an endogenous H_2_S donor, in part ameliorates inflammatory hepcidin and hypoferremia by inhibiting IL-6/JAK2/STAT3 pathway.

### SPRC improves turpentine-induced AI in vivo

**Fig 3:**
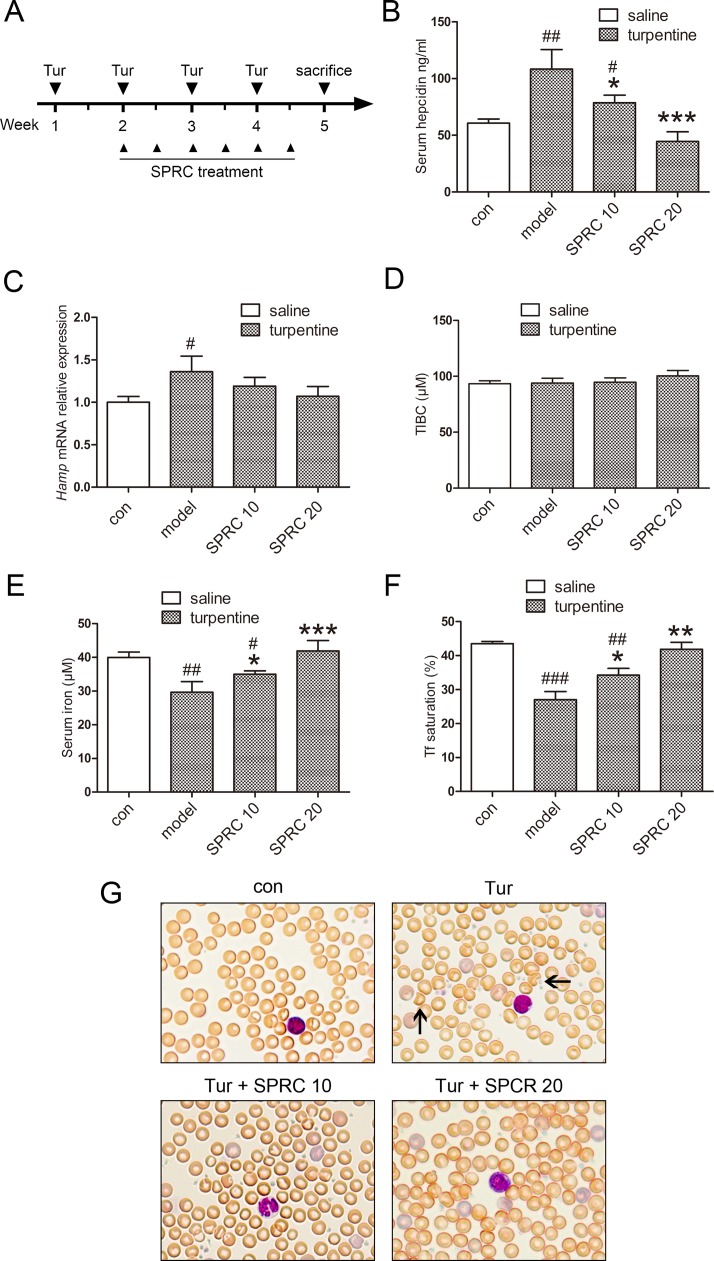
SPRC improves turpentine-induced AI *in vivo*. (A) A diagram about the induction of AI by turpentine and the application of SPRC. (B-C) SPRC treatment ameliorated serum hepcidin levels in turpentine-induced AI model, and a similar trend was observed in hepatic hepcidin mRNA expression (n = 7). (D-F) Total iron binding capacity (TIBC) was unchanged, while serum iron levels and transferrin (Tf) saturation were increased by SPRC (n = 7). (G) Representative images of blood smears with Wright-Giemsa staining showed that red blood cell morphology was improved by SPRC. Solid arrows indicate damaged erythrocytes. Data are presented as the mean ± SEM. ^##^ *p* < 0.01, ^###^ *p* < 0.001 compared with the control group; * *p* < 0.05, ** *p* < 0.01, *** *p* < 0.001, compared with the model group.

We next investigated whether SPRC could treat chronic AI. Turpentine has been widely used to induce inflammatory models for decades, including normocytic, normochromic anemia which shares the same nature clinically [24–26]. The work flow is demonstrated in Fig 3A. As was observed in LPS model, SPRC decreased serum hepcidin levels in the turpentine model (Fig 3B). Although the hepatic hepcidin mRNA levels were relatively low, which might be attributed to the late detection time from the last turpentine injection as reported previously [24], there remained a decreasing trend in the SPRC groups (Fig 3C). No significant change was observed in total iron binding capacity (Fig 3D). As to serum iron and transferrin (Tf) saturation, both doses of SRPC exerted similar treatment effects while SPRC 20 was more pronounced (Fig 3E and 3F). To further assess the effects of SPRC on hemogram, complete blood count was conducted. As illustrated in Table 1, SPRC increased erythrocyte number, hemoglobin content, in addition to hematocrit levels. By performing blood smears and Wright-Giemsa staining, we found that SPRC improved erythrocyte membrane regularity (Fig 3G). These results suggest that SPRC successfully relieves turpentine-induced AI symptoms.

**Table 1:** Hematological and mouse indices.

		Turpentine		
Parameter	con	model	SPRC 10	SPRC 20
RBC (×10^12^/L)	9.86 ± 0.21	7.94 ± 0.55^**###**^	9.02 ± 0.56*	9.10 ± 0.67**
HGB (g/L)	138 ± 3	110 ± 9^**###**^	122 ± 9*	126 ± 11*
HCT (%)	44.3 ± 0.5	36.1 ± 2.8^**###**^	40.3 ± 3.0*	41.2 ± 3.6*
MCV (fL)	45.5 ± 0.6	46.5 ± 1.2	46.2 ± 0.5	47.2 ± 1.3
MCH (pg)	14.1 ± 0.1	13.7 ± 0.5	13.8 ± 0.3	14.0 ± 0.2
PLT (×10^9^/L)	1263 ± 118	1557 ± 378	1857 ± 217	1442 ± 221
RDW-SD (fL)	25.9 ± 0.4	31.1 ± 3.7	29.7 ± 1.4	31.9 ± 5.5
Neutrophil (×10^9^/L)	1.18 ± 0.59	2.28 ± 1.39	1.66 ± 1.14	1.01 ± 0.17*
Lymphocyte (×10^9^/L)	5.79 ± 2.27	4.45 ± 1.21	3.94 ± 1.72	3.58 ± 1.78
Monocytes (×10^9^/L)	1.22 ± 0.36	2.18 ± 1.61^**#**^	2.14 ± 0.85	3.45 ± 3.64
Mouse (g)	26.0 ± 1.0	26.0 ± 1.6	25.3 ± 0.6	26.2 ± 1.8

RBC, red blood cell; HGB, hemoglobin; HCT, hematocrit; MCV, mean corpuscular volume; MCH, mean corpuscular hemoglobin; PLT, platelet; RDW, red blood cell distribution width. Data are presented as the mean ± SD.

^#^ *p* < 0.05

^###^ *p* < 0.001 compared with the control group

* *p* < 0.05

** *p* < 0.01, compared with the model group.

### SPRC ameliorates turpentine-induced AI by blocking IL-6/JAK2/STAT3 pathway

**Fig 4:**
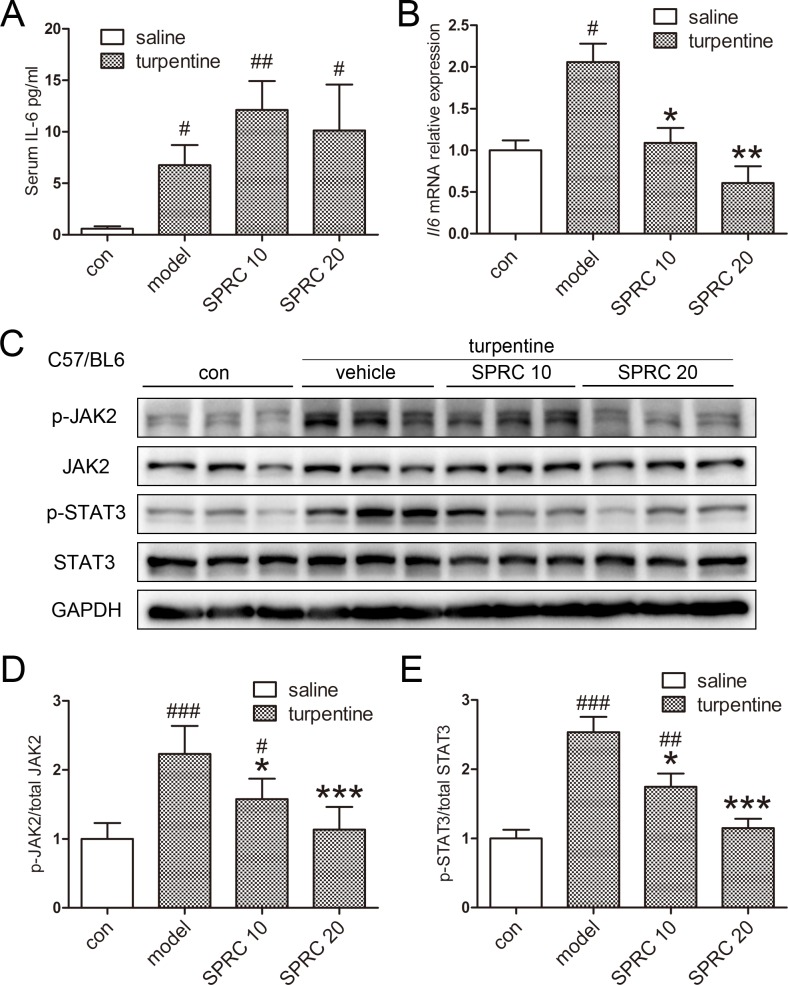
SPRC improved AI by reducing hepatic IL-6/JAK2/STAT3 activation. (A) Serum IL-6 content was not significantly changed by SPRC (n = 7). (B) Hepatic Il-6 mRNA levels were suppressed by SPRC, though only 2-fold change was observed in the model group (n = 7). (C) SPRC successfully ameliorated hepatic JAK2/STAT3 phosphorylation in the turpentine model. (D-E) Densitometry analysis of Fig 5C. Representative immunoblots are presented. Whole uncropped images of Fig 4C are shown in S2 Fig. Data are presented as the mean ± SEM. ^#^ *p* < 0.05, ^###^ *p* < 0.001 compared with the control group; * *p* < 0.05, ** *p* < 0.01, *** *p* < 0.001 compared with the model group.

The results above prompted us to ask whether IL-6/STAT3 pathway was inhibited by SPRC in AI model. As presented in Fig 4A and 4B, both serum IL-6 content and hepatic *Il-6* mRNA levels were induced, but in a much smaller extent than that in the LPS model. Although serum IL-6 was not significantly changed by SPRC, hepatic *Il-6* mRNA expression was suppressed, as was observed previously. Accordingly, hepatic JAK2/STAT3 activation was suppressed by SPRC treatment (Fig 4C–4E). Taken together, we conclude that SPRC improves turpentine-induced AI by inhibiting IL-6/JAK2/STAT3 pathway.

### SPRC ameliorates splenomegaly and reduces splenic iron accumulation during AI

**Fig 5:**
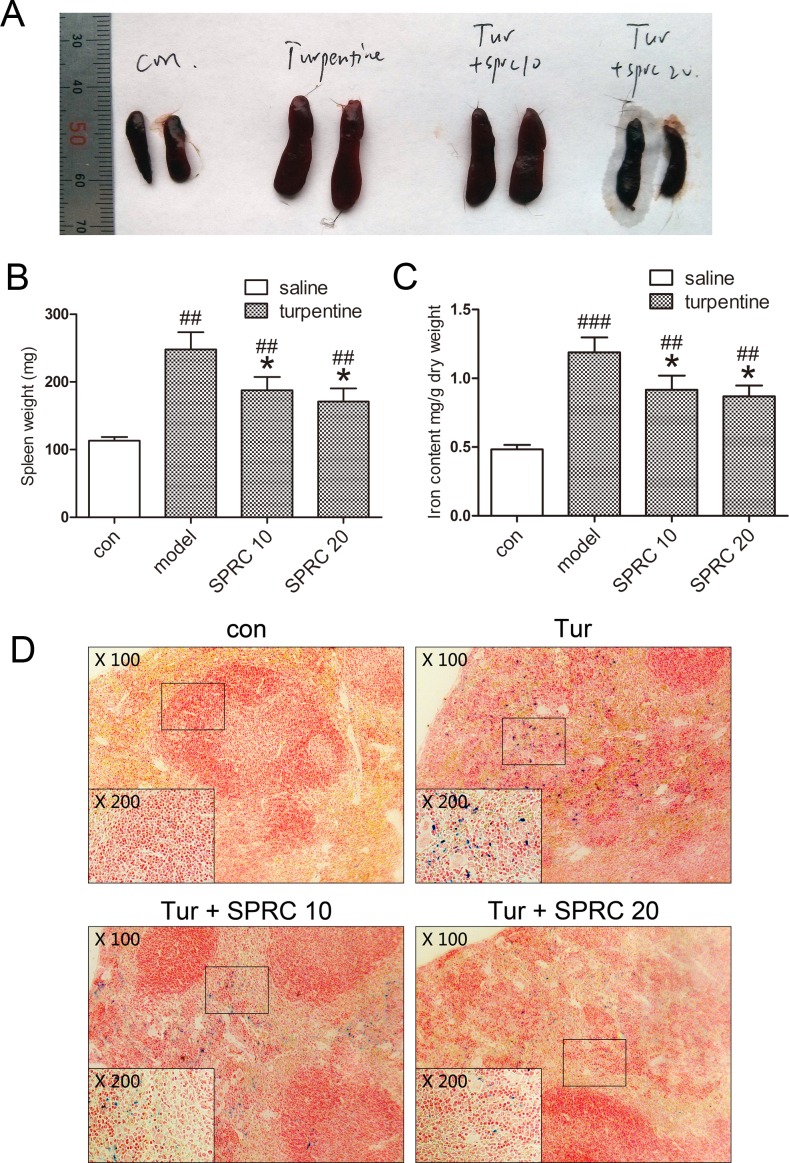
SPRC reduces splenomegaly and corrects splenic iron accumulation in chronic AI model. (A) Representative images indicated relieved splenomegaly by SPRC. (B) Consistent results were observed with mouse spleen weight (n = 7). (C-D) Splenic iron accumulation was relieved by SPRC, as assessed by non-heme iron analysis and Perl’s Prussian blue staining (n = 7). Representative images are presented. Data are presented as the mean ± SEM. ^##^ *p* < 0.01, ^###^ *p* < 0.001 compared with the control group; * *p* < 0.05 compared with the model group.

The spleen plays an important role in immune response, and chronic inflammation is often accompanied by splenomegaly. On the other hand, besides reduced circulating iron level, dysregulation of splenic iron is another hallmark of AI [1]. Thus we turned attention to the effects of SPRC on spleen during chronic AI. As manifested in Fig 5A and 5B, SPRC partially relieved splenomegaly induced by turpentine. Moreover, tissue iron determination indicated that splenic iron accumulation was in part reversed by SPRC (Fig 5C). Similar results were obtained by Perl’s Prussian blue staining with mouse spleen sections (Fig 5D).

## Discussion

In the present study, we demonstrated that SPRC, a novel water-soluble H_2_S donor, exerted inhibition on inflammatory hepcidin. Moreover, SPRC not only relieved hypoferremia induced by acute inflammatory insult, but also improved chronic AI symptoms *in vivo*. Mechanism study indicated that the effects of SPRC were related to decreased IL-6 production, reduced inflammation, and suppressed hepatic JAK2/STAT3 activation. Our results provide new insights into the anti-inflammatory property of H_2_S donors, and suggest new approach for drug development against AI.

AI is one of the most common complications in patients with chronic inflammation. It is estimated that the prevalence of AI is 18–95% in infections, 30–77% in cancer, and 8–71% in autoimmune diseases [1]. However, AI is often unrecognized thus needs appropriate intervention and specific care [27, 28]. Mounting evidences have indicated the relation between anemia and increased cardiovascular risk, higher mortality and poor prognosis [29, 30]. Moreover, normalization of anemia is associated with improved quality of life [31]. Nevertheless, conventional therapies for AI have raised clinical concerns, such as infection risk induced by iron supplement and increased mortality in cancer patients on erythropoietic stimulating agents [32, 33], illustrating an urgent need for alternative remedy of AI. In our study, we identified SPRC, a sustained-releasing H_2_S donor, suppressed inflammatory hepcidin and acted as a potential therapeutic compound for AI. SPRC successfully relieved iron disturbance and reduced hemoglobin levels *in vivo*, the two hallmarks of AI. More work is needed to assess the druggability of SPRC.

Hepcidin, initially named after its antimicrobial property, was first discovered in human urine [34]. Following studies reveal its critical role in iron balance and its regulation by IL-6/STAT3 pathway [9, 35]. During inflammation, phospho-STAT3 dimers directly bind to the promoter region of hepcidin, initiating its transcription [35]. By promoting the degradation of ferroportin, the prominent cellular iron exporter, hepcidin cuts down dietary iron absorption, induces iron retention within liver and spleen, and reduces circulating iron levels. It has been well demonstrated that hepcidin plays the key role in the development of AI [6], making it an ideal therapeutic target for iron-restrictive anemia. In principle, hepcidin antagonists either suppress hepcidin expression induced by upstream signaling, or inhibit iron-regulating effects triggered by hepcidin. For the former strategy, it is particularly effective to decrease cytokine production, typically IL-6, and block related signaling pathway. Indeed, several studies have focused on bone morphogenetic proteins or IL-6 pathways and identified some potential hepcidin antagonists [36–38]. In accordance, our results demonstrated SPRC as a potent inhibitor of IL-6 production and hepcidin activation, making it a strong candidate for hepcidin antagonists.

H_2_S used to be regarded as a noxious gas, until recent recognition as the third gasotransmitter. Cystathionine γ-lyase (CSE) and cystathionine-β-synthase (CBS) are two major endogenous H_2_S synthases mainly expressed in heart and brain. Incremental studies have reported the involvement of H_2_S in cardiovascular system, central nervous system, and inflammation [11, 39, 40]. Several researches from independent groups have indicated the protective property of H_2_S by preserving mitochondrial function during myocardial infarction [41, 42]. Gong et al. suggests that H_2_S attenuates lipopolysaccharide-induced cognitive impairment in rats [43]. In accordance with our previous observation that NaHS suppresses inflammation and reduces IL-6 secretion, Whiteman et al. claims reduced IL-6 production by H_2_S application [15], which is probably attributed to inhibition of NF-κB [44]. On the other hand, very few studies are available regarding H_2_S and iron metabolism. In our recent work, we demonstrates that NaHS, an exogenous H_2_S donor, inhibits hepcidin and relieves hypoferremia induced by LPS [14]. Consistently, SPRC, a CSE-dependent endogenous H_2_S donor, showed similar effects *in vivo*, supporting the anti-inflammatory effects of H_2_S.

As an H_2_S donor, SPRC shows multiple regulation in different disease models. A number of studies have revealed its therapeutic potential in cardiovascular system [20, 45] and inflammation [22, 46]. Consistent with previous observations, here we demonstrated corrected iron disturbance and relieved AI symptoms by SPRC. Our work broads the potential application of SPRC, and provides new evidence of the gasotransmitter role of H_2_S.

## Conclusions

SPRC, a novel H_2_S donor, relieves hypoferremia and anemia both in acute and chronic models of inflammation. The effects of SPRC were attributed to inhibited hepatic JAK2/STAT3 activation and reduced hepcidin production. Our results provide new insights into the anti-inflammatory property of H_2_S, and suggest SPRC as a potential remedy against AI.

## Supporting Information

S1 Fig Whole uncropped images of the original western blots for Fig 2D. Whole uncropped images of the original western blots for p-JAK2 (A), JAK2 (B, ten lanes from the right are used), p-STAT3 (C), STAT3 (D), and GAPDH (E) in Fig 2D.
(TIF)

S2 Fig Whole uncropped images of the original western blots for Fig 4C. Whole uncropped images of the original western blots for p-JAK2 (A), JAK2 (B, ten lanes from the right are used), p-STAT3 (C), STAT3 (D), and GAPDH (E) in Fig 4C.
(TIF)
