# Supplementary figures and images for: S-Propargyl-Cysteine, a Novel Hydrogen Sulfide Donor, Inhibits Inflammatory Hepcidin and Relieves Anemia of Inflammation by Inhibiting IL-6/STAT3 Pathway
Source: PLoS One. 2016 Sep 20;11(9):e0163289. doi: 10.1371/journal.pone.0163289 (PMC5029915; doi:10.1371/journal.pone.0163289)

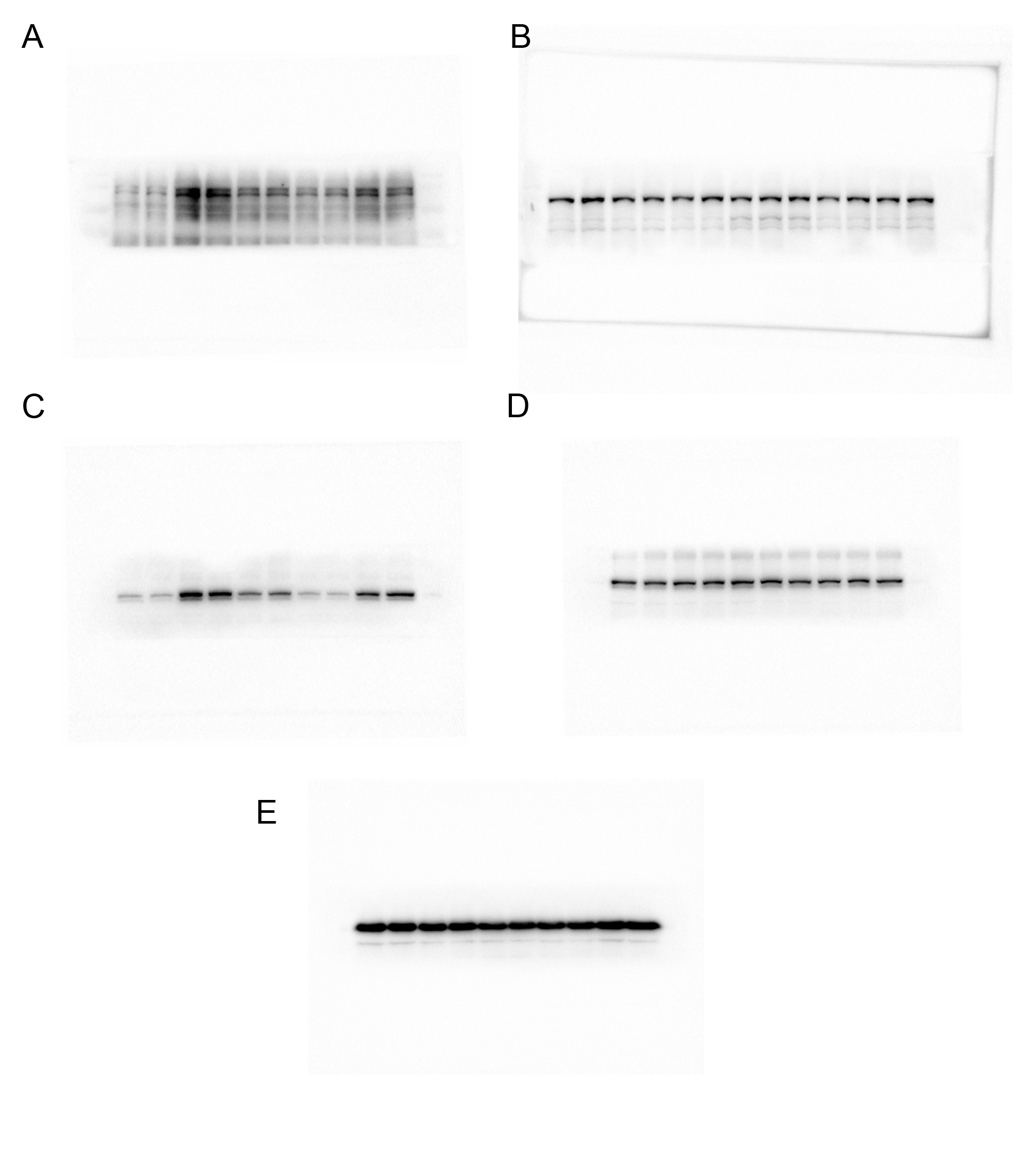

Supplement: S1 Fig — Whole uncropped images of the original western blots for p-JAK2 (A), JAK2 (B, ten lanes from the right are used), p-STAT3 (C), STAT3 (D), and GAPDH (E) in Fig 2D. (TIF) [file pone.0163289.s001.tif]

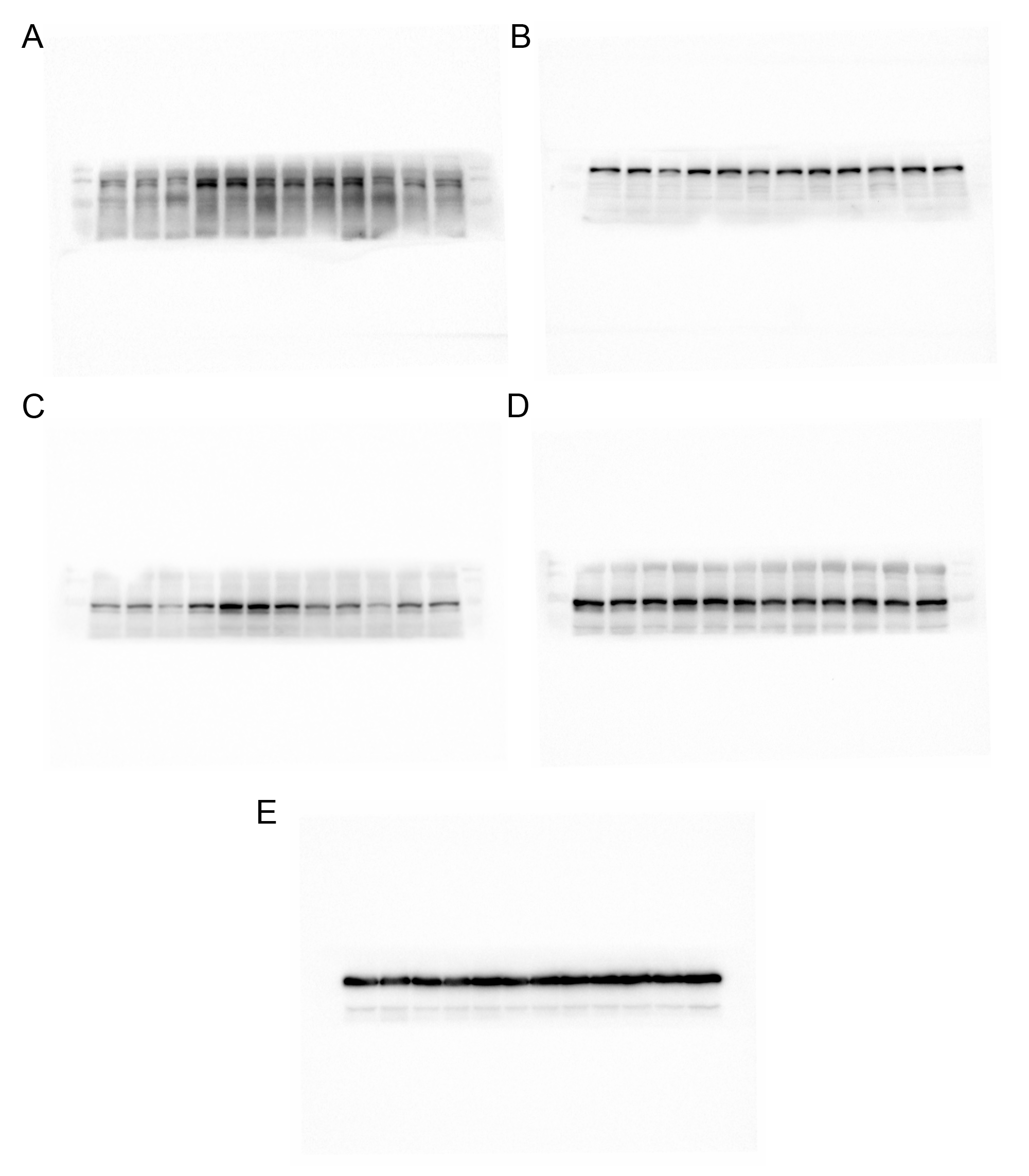

Supplement: S2 Fig — Whole uncropped images of the original western blots for p-JAK2 (A), JAK2 (B, ten lanes from the right are used), p-STAT3 (C), STAT3 (D), and GAPDH (E) in Fig 4C. (TIF) [file pone.0163289.s002.tif]
